# Three-dimensional ultrastructure of *Plasmodium falciparum* throughout cytokinesis

**DOI:** 10.1371/journal.ppat.1008587

**Published:** 2020-06-08

**Authors:** Rachel M. Rudlaff, Stephan Kraemer, Jeffrey Marshman, Jeffrey D. Dvorin

**Affiliations:** 1 Biological and Biomedical Sciences, Harvard Medical School, Boston, Massachusetts, United States of America; 2 Division of Infectious Diseases, Boston Children’s Hospital, Boston, Massachusetts, United States of America; 3 Center for Nanoscale Systems, Harvard University, Cambridge, Massachusetts, United States of America; 4 Carl Zeiss Microscopy, LLC, Thornwood, New York, United States of America; 5 Department of Pediatrics, Harvard Medical School, Boston, Massachusetts, United States of America; University of Texas Southwestern Medical School, UNITED STATES

## Abstract

New techniques for obtaining electron microscopy data through the cell volume are being increasingly utilized to answer cell biologic questions. Here, we present a three-dimensional atlas of *Plasmodium falciparum* ultrastructure throughout parasite cell division. Multiple wild type schizonts at different stages of segmentation, or budding, were imaged and rendered, and the 3D structure of their organelles and daughter cells are shown. Our high-resolution volume electron microscopy both confirms previously described features in 3D and adds new layers to our understanding of *Plasmodium* nuclear division. Interestingly, we demonstrate asynchrony of the final nuclear division, a process that had previously been reported as synchronous. Use of volume electron microscopy techniques for biological imaging is gaining prominence, and there is much we can learn from applying them to answer questions about *Plasmodium* cell biology. We provide this resource to encourage readers to consider adding these techniques to their cell biology toolbox.

## Author summary

*Plasmodium falciparum* is the parasite that causes the most severe form of human malaria. The signs and symptoms of malaria result from exponential expansion in the number of parasites occupying red blood cells during the blood stage of infection. At this stage, the parasite uses a non-canonical method of replication known as schizogony. In schizogony, the parasite produces the components for 20–36 daughter cells in a shared cytoplasm through asynchronous rounds of nuclear and organellar replication. At the end of schizogony, a single synchronous round of cell division produces individual invasive daughter cells known as merozoites. We use advanced electron microscopy techniques to evaluate the stages of this process at high resolution in three dimensions. Our data illustrate the complex three-dimensional geometry of merozoite formation and provide new insights about the relative synchrony of these events.

## Introduction

**Fig 1:**
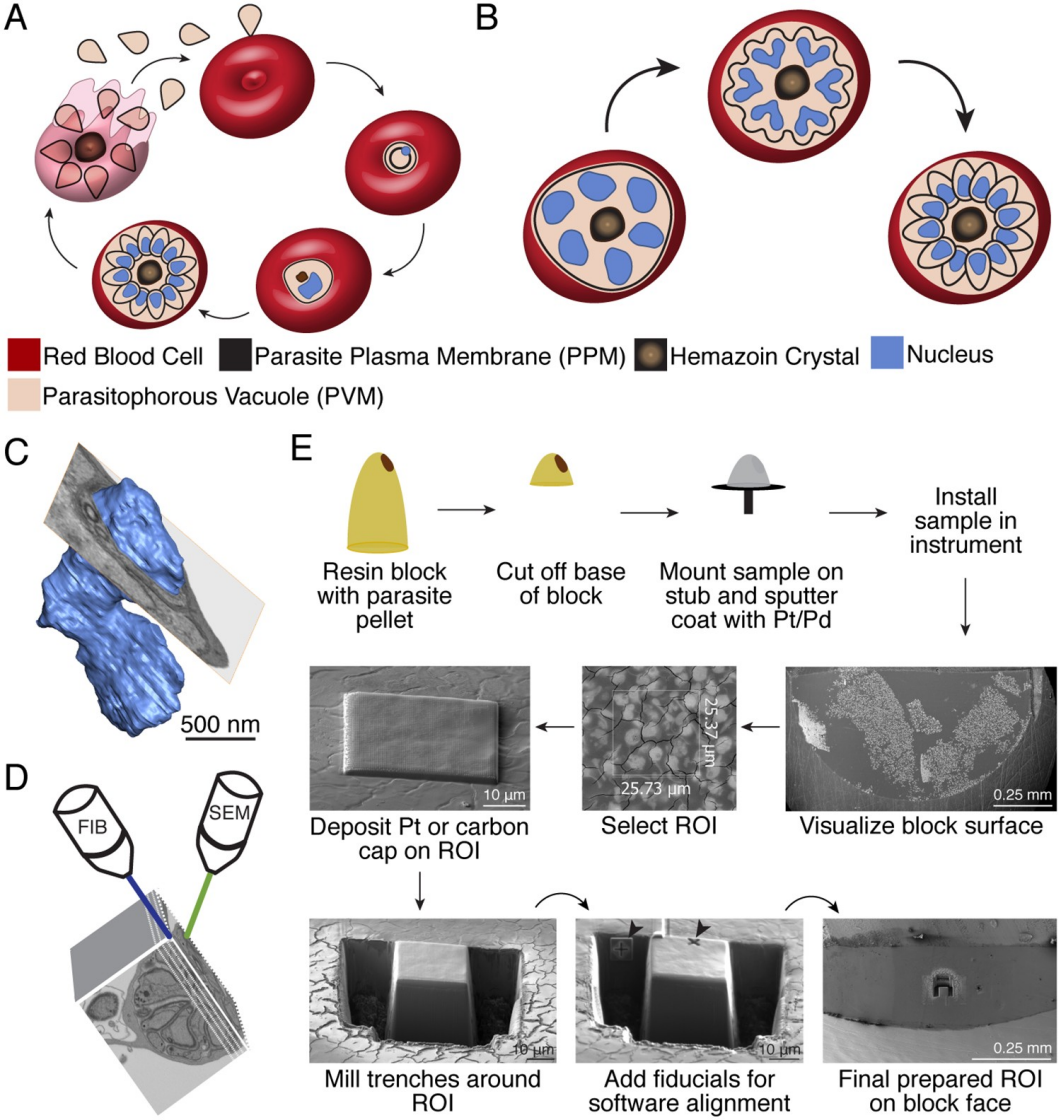
*Plasmodium falciparum* asexual life cycle and FIB-SEM sample preparation. **A.** A merozoite invades a red blood cell (RBC) to initiate infection (top center). Post-invasion, the parasite develops into a biconcave early trophozoite known as a ring inside its parasitophorous vacuole (PV) (right). The parasite becomes more metabolically active and begins to replicate its DNA as a late trophozoite (bottom right). As a schizont (bottom left) the parasite produces nuclei and organelles for its daughter cells, then undergoes cytokinesis to package these contents into individual merozoites. Egress (left) releases merozoites from the PV and RBC to invade a new RBC. **B.** In schizogony, the parasite produces DNA and organelles for its daughter cells asynchronously within a common cytoplasm (left). During segmentation, the parasite plasma membrane invaginates around nascent daughter cell buds (middle) to form fully segmented merozoites (right). This model depicts the hypothesis that the final round of karyokinesis is synchronous and simultaneous with cytokinesis. **C.** Volume electron microscopy reveals features not captured in single slices. A slice of EM data shown as an ortho-slice through the rendered nucleus, which contains subsurface features unseen in the single slice shown. Scale bar interpolated from structure sizes in EM data **D.** FIB-SEM employs two beams to serially mill the face off a resin block with a focused ion beam, then image with a scanning electron beam. In some instruments, milling and imaging can be performed simultaneously. **E.** FIB-SEM workflow: A resin block (yellow) with embedded parasite pellet (brown) is shaved down to reduce block height, mounted on a SEM stub with carbon tape, and sputter coated with platinum-palladium (Pt/Pd). The sample is installed in the FIB-SEM instrument and block surface visualized to identify regions of cell density. A 1–2 μm platinum (shown) or carbon (for data captured in this resource) cap is applied to the region of interest (ROI) to improve conductivity. Trenches are milled to expose the front of the block face for imaging and fiducials (arrows) are milled for focusing by the Atlas control software. For the data presented in this resource, fiducials were instead sandwiched between layers of the carbon cap.

Infection by the eukaryotic parasite *Plasmodium falciparum* causes the most severe form of human malaria. During the asexual blood stage of the *Plasmodium falciparum* life cycle, when the signs and symptoms of human malaria are manifest, the parasites inhabit red blood cells where they grow and produce progeny in the unique intraerythrocytic development cycle[1]. This cycle is initiated when a merozoite invades a red blood cell (RBC), enveloping itself in the parasitophorous vacuole then developing into a biconcave early trophozoite-stage parasite known as a ring (Fig 1A). Shortly after invasion, the parasite begins exporting proteins to remodel the RBC architecture. The parasite develops into a late-stage trophozoite as it feeds on the RBC, grows, and begins replicating its DNA and organelles while continuing to remodel the RBC[2]. Once the parasite has three or more nuclei, it is known as a schizont (Fig 1A and 1B). During the schizont stage, the *P*. *falciparum* parasite ramps up replication–producing nuclei for approximately 16–36 daughters in multiple rounds of nuclear division and expanding its mitochondrion and non-photosynthetic remnant chloroplast, the apicoplast, to a serpentine structure with branches spanning the growing cell[3]. While the early rounds of nuclear division (karyokinesis) are asynchronous[4], the final round of karyokinesis has been hypothesized to occur synchronously and simultaneously with cytokinesis[5]. In the final stages of schizogony, the parasite orchestrates segmentation–generating structural scaffolds that define the apical end for each emerging daughter cell, dispensing vesicles likely derived from the nuclear membrane[6] or endoplasmic reticulum (ER) that will form the apical organelles (rhoptries, micronemes, and dense granules), dividing its apicoplast then mitochondrion[3], completing nuclear division, and finally directing formation of the structural inner membrane complex and plasma membranes around each daughter of the syncytium. Rupture of the parasitophorous vacuolar membrane followed by rupture of the RBC plasma membrane then frees these invasive daughter cells to reinitiate the asexual life cycle.

Our knowledge of this intricate process has been formed to a great extent by cell biologic analysis–in particular by electron and immunofluorescence microscopy–of maturing parasites. This is despite the fact that throughout the asexual life cycle, *Plasmodium* parasites range from as small as 1.2–1.5 μm as merozoites to as large as 6 μm as schizonts (current data and [7]). The attainable resolution of standard fluorescent microscopy is defined by Abbe diffraction equation (resolution = λ / (2 * NA), where λ is the wavelength of light used–typically 350–700 nm–and NA is the numerical aperture of the objective–typically <1.5). Practically, this limits the amount of information gained from immunofluorescence microscopy to a resolution of ~200 nm, a resolution that does not generally allow separation of structures smaller than this size. In the past two decades, multiple super-resolution techniques[8–12] have improved this resolution approximately 2- to 5-fold (reviewed in [13]). However, even when utilizing these super-resolution techniques, the ability of light microscopes to delineate small structures within the parasite is limited. Therefore, there is a storied tradition in the field of utilizing electron microscopy (EM) to parse out the specifics of *Plasmodium* ultrastructure. These studies, largely performed on single or reconstructed serial sections, have formed our perspective on this important parasite.

EM has been an essential tool for visualization of *Plasmodium* ultrastructure even prior to the development of *in vitro* culture methods for the parasite[14]. Due to the smaller wavelength of electrons compared to light, image resolution can reach the single digit nanoscale, allowing visualization of structures indistinguishable with optical techniques–even with super-resolution light microscopy, structures such as the rhoptry neck and apical ring have thus far been indistinguishable, and organelles are often only visible as clusters of pixels. Additionally, electron micrographs feature a high information density, rendering many cellular components visible simultaneously with the proper combination of staining agents, as compared to fluorescence microscopy which, while highly sensitive and specific for individual proteins, can only provide spatial context for a few at a time.

Given the superior resolution of EM, its primary drawback has been that information must be inferred from single sections or laboriously reconstructed serial sections. Single sections, while informative, do not provide details about the state of the entire cell volume (Fig 1C), which can be particularly important for the asexual blood stage described in this report. It can also be particularly challenging to interpret data on mutant parasites from single slices. However, obtaining volume information from EM has typically required skillful sectioning, imaging, and alignment of 50 or more thin sections or requires stitching together data from multiple serial electron tomograms to ultimately reconstruct a single parasite[15–23].

Recently, the application of two volume electron microscopy methods, Serial Block Face–Scanning Electron Microscopy (SBF-SEM) [24, 25] and Focused Ion Beam–Scanning Electron Microscopy (FIB-SEM) [26], to parasitology have allowed improved visualization of the three dimensional ultrastructure of *Plasmodium* parasites, largely by automating the processes of sectioning and visualization. In SBF-SEM a microtome is housed inside of an SEM and used to consecutively shave off a thin surface layer (generally ~50 nm) of a parasite-embedded resin block exposing new structural features on the block surface, which are then imaged by SEM. This process is repeated serially to generate data through the cell volume. In the past decade, SBF-SEM has been utilized to examine *P*. *falciparum* MS822 parasites through their asexual life cycle [27], to characterize *P*. *knowlesi* ultrastructure across its entire life cycle [28], to illustrate the maturation of *P*. *falciparum* NF54 gametocytes from stage I through stage V, as well as the consequences of a PfPHIL1 knockdown on this stage [29], and to demonstrate that protein prenylation is required for mature digestive vacuole formation [30].

Similarly, Focused Ion Beam–Scanning Electron Microscopy (FIB-SEM), a technique originally developed for materials science applications, has also been gaining increasing prominence for three-dimensional visualization of biological samples[26, 31–33]. In this technique, a focused ion beam, in most current instruments Gallium, is used to mill a plastic-embedded sample with nanoscale precision and expose sub-surface regions for imaging by SEM (Fig 1D and 1E). Like SBF-SEM, serial milling and imaging is performed to generate data through the cell volume, but unlike SBF-SEM, by milling with a FIB one can achieve z-slices as thin as 4 nm [34]. FIB-SEM has been utilized to analyze nuclear pore number and positioning across the asexual life cycle in *P*. *falciparum* NF54 parasites [35], to examine *P*. *chaubadi* 3D ultrastructure, and in particular, the shape of the *P*. *chaubadi* tubulovesicular network and hemoglobin-containing tubules [36], to examine *P*. *cyanomolgi* oocyst formation [37], and to demonstrate the aberrant morphology of schizonts upon knockdown of PfCINCH, an essential basal complex protein [38].

In this report, we utilize FIB-SEM serial sections of *P*. *falciparum* 3D7 parasites to describe the process of segmentation in schizogony (Fig 1B). In order for viable daughter cells to be produced, this process must occur with high fidelity–yielding merozoites that each contain a single nucleus (with associated centrosome and ER), mitochondrion, and apicoplast. Furthermore, segmentation must produce polarized cells with an apical end, defined by the presence of an apical ring, rhoptries, and associated small, electron-dense organelles required for invasion, and a basal end defined by the presence of a basal ring. During the course of segmentation nascent merozoites become cloaked with the parasite plasma membrane (PPM), which is thought to originate from the invaginating mother cell membrane, as well as the underlying inner membrane complex (IMC), which is built along the inside edge of the PPM as segmentation progresses.

Here, we examine the three-dimensional ultrastructure of each of these categories of cellular features (membranes, nuclei, mitochondria/apicoplast, apical/basal ends) individually over multiple stages of segmentation to produce an updated view of *Plasmodium falciparum* budding. For each stage, we provide a 3D-rendering as well as the series of sections used to generate it. Importantly, these cells are snapshots of the kinetic growth process of parasite segmentation. Therefore, while they illustrate the major known features of segmentation, there are likely many intermediates not encompassed here. We hope that this atlas provides a renewed appreciation of the three-dimensional ultrastructure of this fascinating and complex stage of *P*. *falciparum* development.

## Results

### Parasite data and visualization

We performed FIB-SEM experiments on samples of wild type (3D7) *P*. *falciparum* parasites synchronized to 46–48 hours post invasion (hpi) and 48–50 hpi. The 48–50 hpi sample was treated with the cysteine protease inhibitor E64 for 3 hours prior to collection. Parasites imaged from the [–]E64 46–48 hpi block are labeled alphabetically and early segmentation parasite A and B and mid-segmentation parasite A and B were rendered (S1 Table). Parasites imaged from the [+]E64 48–50 hpi block are labeled numerically. Early segmentation parasites 1 and 2, mid-segmentation parasites 1 and 2, a parasite at PVM rupture and a parasite post-PVM rupture were rendered (S1 Table). The raw data for these parasites is available, as well as the raw data for a [–]E64 post-PVM rupture parasite at the Electron Microscopy Public Image Archive (EMPIAR)[39] (EMPIAR-10392 at https://www.ebi.ac.uk/pdbe/emdb/empiar/entry/10392/).

### Plasma membrane dynamics across cytokinesis

**Fig 2:**
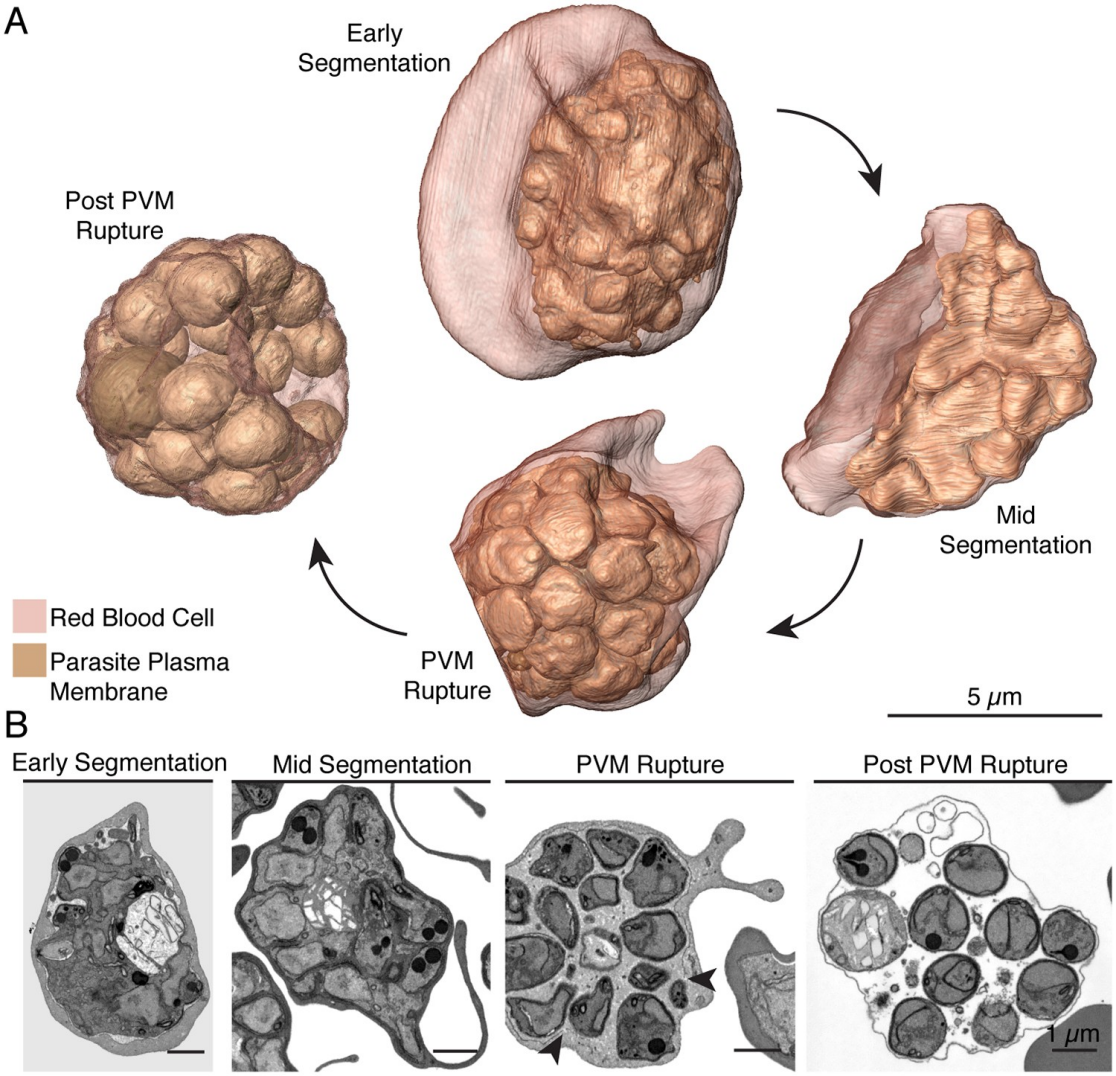
Parasite plasma membrane dynamics throughout segmentation. **A.** Renderings of the four different schizont stages inside their host red blood cells through segmentation. Scale bars of renderings interpolated from structure sizes in EM data. At early segmentation, the mother parasite plasma membrane begins to invaginate, forming buds for each nascent merozoite that slightly protrude from the mother parasite mass. At mid-segmentation, these buds are more pronounced and jut out of the mother parasite. By PVM-rupture, the parasite plasma membrane cloaks each new merozoite, which is shaped like an inverted cone. Post-PVM rupture, merozoites fill the red blood cell and take on a more spherical shape. **B.** Single SEM images of the four schizonts chosen for rendering. We note that for the early segmentation schizont, the increased contrast between the parasite plasma membrane and parasitophorous vacuole is an artifact of sample preparation and provides additional contrast helpful for rendering. Arrows indicate region of PVM rupture.

By electron microscopy, multiple stages of segmentation are visible as buds for the nascent daughter cells form, become more pronounced, and eventually are separated from the residual body as complete merozoites. As segmentation begins (labeled “early segmentation”), our FIB-SEM reconstructions reveal the plasma membrane of the mother parasite begins to invaginate around developing apical buds, which slightly protrude out of the ovoid parasite mass (Fig 2). The signal for and molecular components required for this invagination remain unknown. As budding progresses (labeled “mid segmentation”), membranes push further inward, surrounding the apical organelles and nucleus and encroaching on the undivided interior of the mother cell. Parasite buds grow more pronounced and appear as spires jutting out of the center of the parasite. Finally, as segmentation completes, the membranes pinch off from the food vacuole to enclose merozoites that each contain a full set of organelles. These are shaped like inverted cones, with apices facing out and basal ends facing the interior of the cell. It is important to note that the rendered parasites were fixed and dehydrated prior to resin embedding. Thus, the exact shape of the membranes is likely affected by this procedure. These potential artifacts may be less prominent in EM studies of parasites prepared with other methods, such as cryo-hydration and sectioning[40]. Merozoites are then released into the red blood cell by parasitophorous vacuolar membrane rupture where they take on an egg-shaped geometry with an apical prominence. A change in merozoite shape upon release has also been noted for *P*. *knowlesi*[28]. When RBC rupture is uninhibited, these daughter cells would be quickly released into the supernatant for reinvasion.

### Nuclear morphology

**Fig 3:**
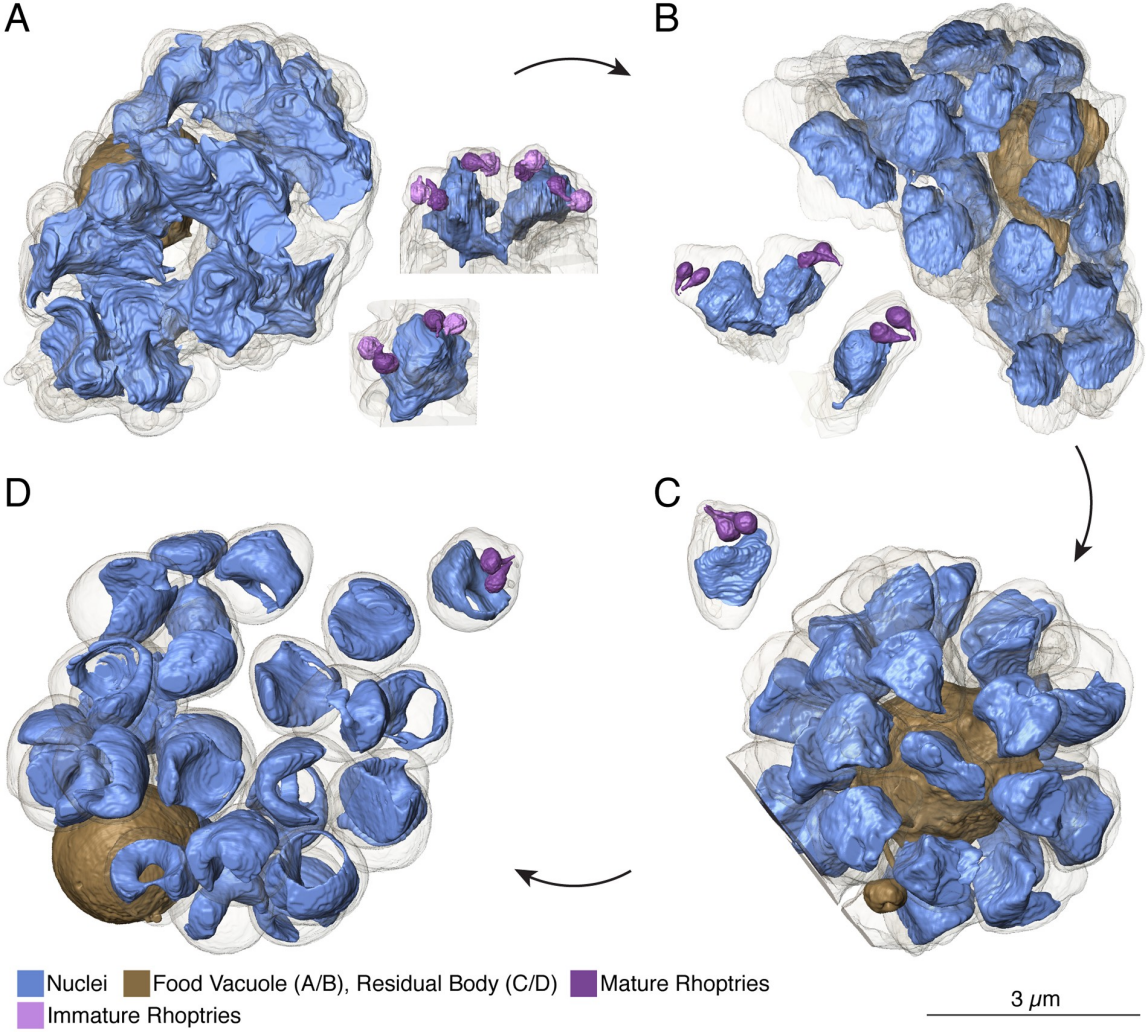
Nuclear architecture throughout segmentation. **A.** In early segmentation, nuclei appear jagged and are associated with two (in 11 of 13) or four (in 2 of 13) sets of rhoptries. The mature rhoptry from each pair is in close association with the nucleus. **B.** In mid-segmentation, nuclei have a smoother appearance, and of fully rendered nuclei, five are 2n and 13 are 1n. Half of imaged rhoptry sets retain a nuclear association. **C.** By PVM rupture, all nuclei are separated into their respective merozoites. No rhoptry sets have a visible nuclear association. **D.** Post-PVM rupture, nuclei have depressed centers, forming a toroid shape or C-shape. We note that this may be due to prolonged RBC entrapment with E64. One daughter cell was ruptured in sample preparation, resulting in two lobes of bridged DNA (seen near the top of the cell). In A and B, the food vacuole is rendered, in C and D, the residual body, containing the food vacuole and residual cytoplasmic material, is rendered. Scale bars of renderings interpolated from structure sizes in EM data.

**Fig 4:**
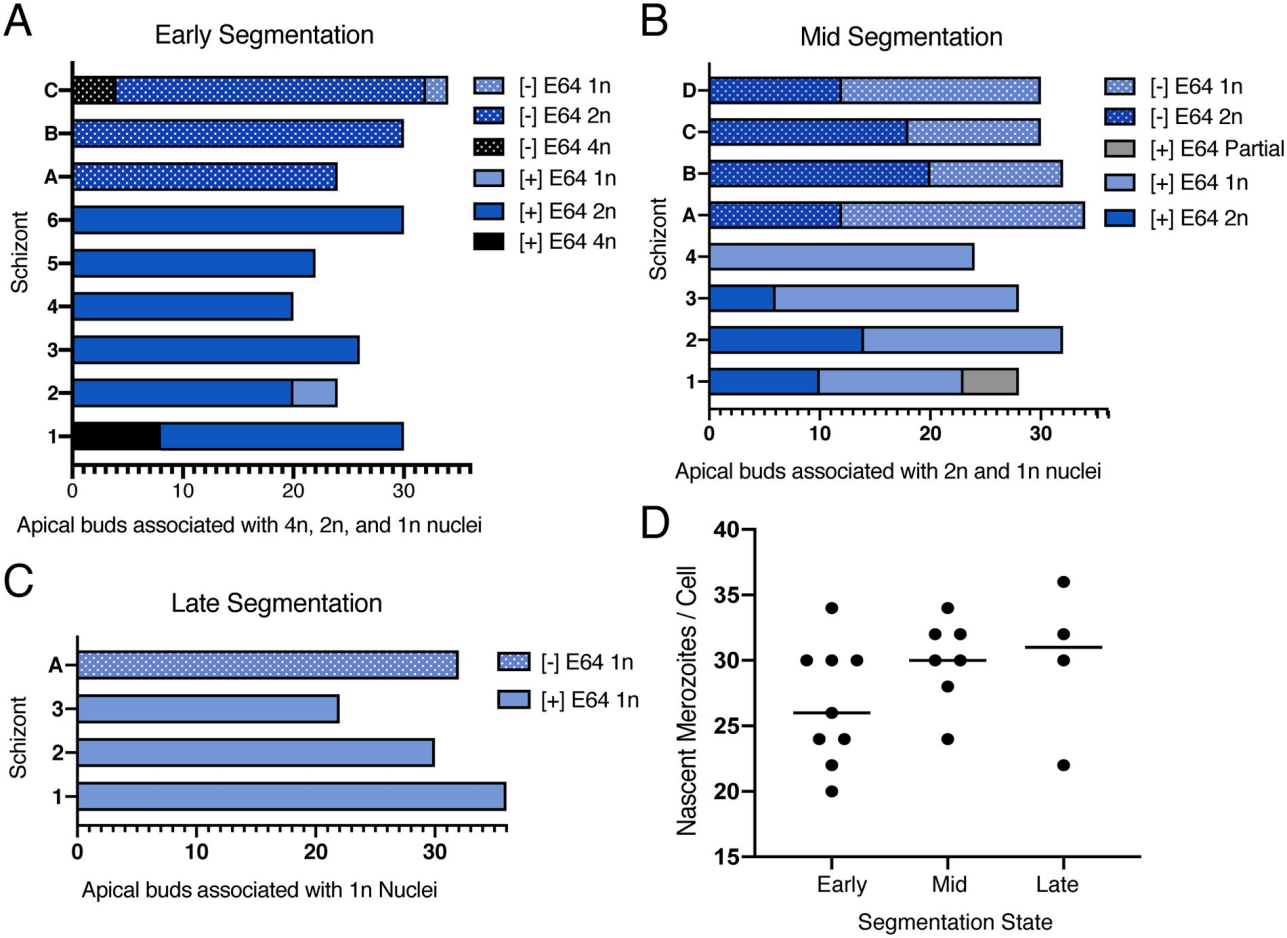
Nuclear status of parasites at early, mid, and late segmentation. **A.** Graph demonstrating the number of apical buds associated with 4n, 2n, and 1n nuclei in three schizonts from an E64-untreated block and six schizonts from an E64 treated block. **B.** Graph demonstrating the number of apical buds associated with 2n and 1n nuclei in four schizonts from a [–]E64 (A-D) and four schizonts from a [+]E64 (1–4) treated sample. **C.** Graph depicting the number of apical buds associated with 1n nuclei in one [–]E64 treated schizont and three [+]E64 treated schizonts. **D.** Plot of the number of nascent merozoites per fully captured cell. Line placed at median for each set.

By the time membrane invagination begins, the nuclear content for each daughter cell has presumably been produced but is uncompacted and not fully divided. Compared to the later stages, the nuclei appear jagged at early segmentation when rendered in 3D (Fig 3A, S1 Figure). Each separate nucleus corresponds to one, two, or four apical buds, which are identified by the presence of early rhoptries (Fig 3A, S1 and S2 Tables, S1 and S2 Figures). Because we have never observed a fully segmented merozoite with more than one set of apical organelles in wild type parasites, the number of apical buds serves as a proxy for the eventual number of fully divided nuclei. Following this logic, a nucleus with two associated apical buds is assumed to be 2n–and will eventually undergo karyokinesis to generate two 1n nuclei prior to the completion of segmentation. In early-segmentation schizont 1, there are eleven 2n and two 4n nuclei, demonstrating asynchrony for the proposed final “synchronized” nuclear division[5]. The asynchrony of karyokinesis is evident in the mid-segmentation schizont as well. By mid-segmentation nuclei are compact and either fully divided into individual nuclei or in the obvious process of splitting from 2n to 1n, with heads of nuclear buds veering away from their connected stalk in a heart shape (Fig 3B). These data demonstrate that the final round of karyokinesis is asynchronous, a finding that becomes readily apparent with volume EM where nuclei and apical structures can be rendered with high precision. In mid-segmentation parasite 1, 13 complete nuclei are fully separated, five are 2n, and five are not fully captured in the image series (Fig 3B). To ensure that this observation was not due to the impact of E64 treatment on nuclear division and to examine this finding in more cells, we segmented nuclei and rhoptries for two early segmentation schizonts (A and B) and two mid-segmentation schizonts (A and B) from an E64-naïve sample. In addition, we rendered the nuclei and rhoptries for an additional E64-treated early segmentation and mid segmentation schizont (for each, schizont “2”). At early segmentation, parasite 2 contained 10 2n and 4 1n nuclei and parasites A and B contained 12 and 15 2n nuclei, respectively (Fig 4A, S1 Table, S1 Figure). At mid segmentation, parasite 2 contained seven 2n and 18 1n nuclei, parasite A contained six 2n and 22 1n nuclei, and parasite B contained 10 2n and 12 1n nuclei (Fig 4B, S1 Table, S1 Figure). For additional confirmation, we counted the number of rhoptry pairs each nucleus was associated with in five additional early segmentation schizonts and all fully captured mid and late segmentation schizonts, in an E64 treated and an E64 untreated sample. At early segmentation, five of the six schizonts contained only 2n nuclei and one contained one 4n, 14 2n, and two 1n nuclei (Fig 4A, S2 Table). At mid segmentation, three of the four schizonts contained both 2n and 1n nuclei, and one schizont contained only 1n nuclei (Fig 4B, S2 Table). At late segmentation, all four schizonts contained only 1n nuclei (Fig 4C, S2 Table). The total number of nascent merozoites per cell for all fully captured early, mid, and late segmentation schizonts are plotted in Fig 4D. At PVM rupture, all nuclei are fully divided and situated in the bottom two-thirds of the merozoite in a rounded rectangular shape (Fig 3C). Following release into the RBC, the nucleus takes on a toroid geometry, with edges pressed along the sides and basal end of the daughter cell and a depression or hole in the center (Fig 3D). This late morphology may result from the unnaturally prolonged entrapment of escaped merozoites in the RBC by the cysteine protease inhibitor E64 used to obtain the images, but we were able to capture a post-PVM rupture parasite whose nuclei exhibited the same geometry in the [–]E64 sample, suggesting that this *bona fide* change in nuclear shape occurs when daughter cells are fully released from the PVM. The data for this parasite was not of high enough quality for 3D rendering, but the raw images can be downloaded from EMPIAR (https://www.ebi.ac.uk/pdbe/emdb/empiar/entry/10392/).

### Apicoplasts and mitochondria

**Fig 5:**
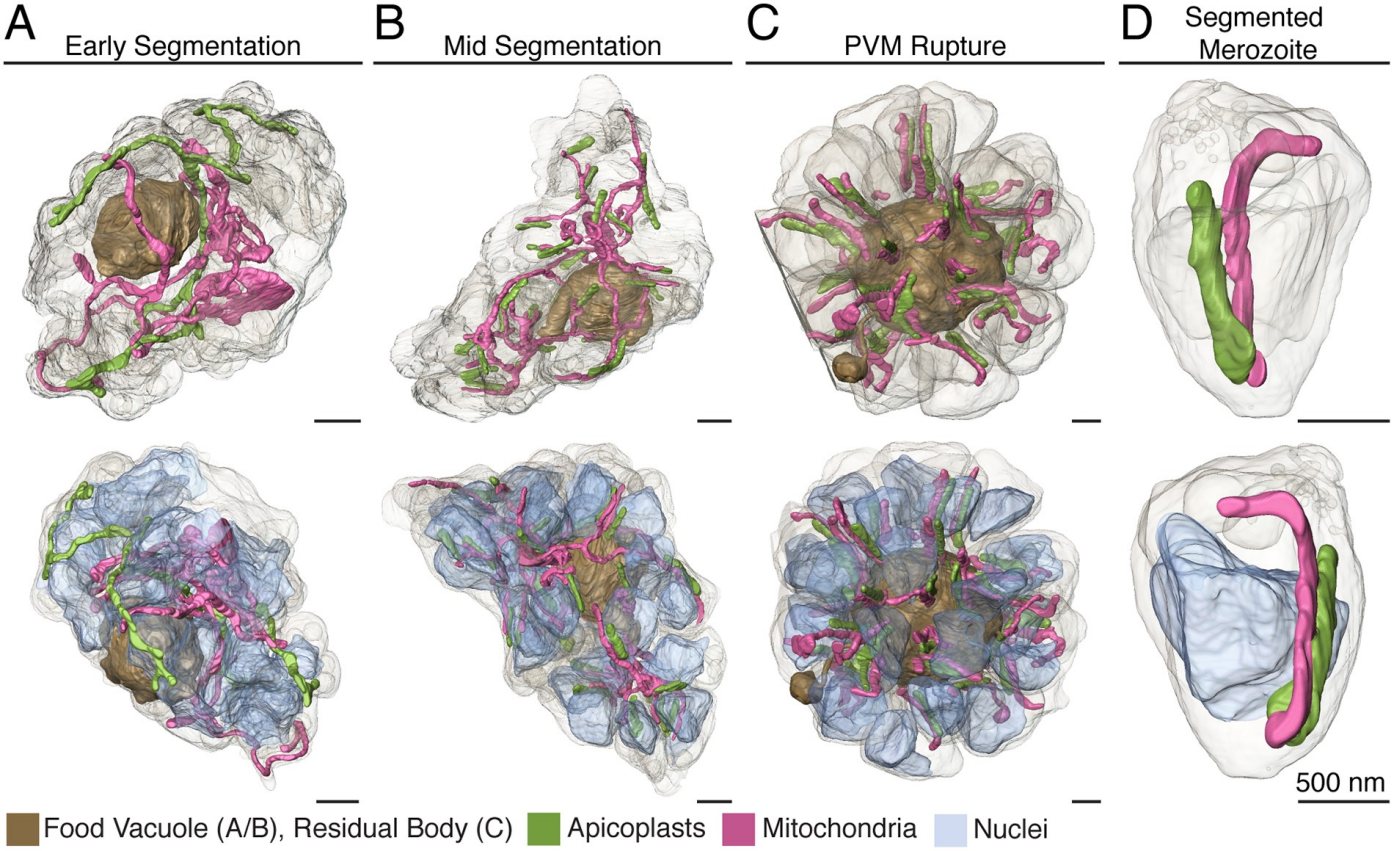
Apicoplast and mitochondria morphology throughout segmentation. **A.** In early segmentation, the apicoplast and mitochondrion each form a sinuous structure throughout the parasite. **B.** By mid-segmentation, apicoplasts have divided to form one organelle for each nascent daughter cell and are closely associated with the mitochondrion, which remains undivided. **C.** At PVM rupture, both apicoplasts and mitochondria are divided and are largely situated within segmented daughter cells. **D.** A segmented merozoite from the schizont in (C), with a single mitochondrion and apicoplast. Scale bars of renderings interpolated from structure sizes in EM data.

Prior to segmentation, the apicoplast and mitochondrion grow and branch without dividing[3], so that in early segmenting schizonts these organelles each form a sinuous structure throughout the parasite. At this stage, they do not reach up into developing buds, but instead curve through the interior of the parasite, winding around the nuclei, which fill much of the available space (Fig 5A, S1 Table). By mid-segmentation, the branched apicoplast divides to produce a daughter organelle for each nascent merozoite (Fig 5B, S1 Table)[27]. Each new apicoplast is then in close spatial proximity to the branched mitochondria, either at one of its poles or along its length. Together, the branched mitochondrion and divided apicoplast reach up into the forming merozoite buds. Finally, in fully segmented merozoites (Fig 5C), mitochondria have also divided into individual daughter organelles. Within each daughter cell the newly formed mitochondrion and apicoplast remain closely approximated along their longitudinal axis and align along one edge of the cell (Fig 5D). Throughout this process the mitochondrion is larger and more tortuous than the apicoplast, and in divided daughter merozoites it is about one third larger than the divided apicoplast, a phenomenon also observed in volume EM data produced in other studies[27, 38]. Apicoplasts and mitochondria were differentiated in the data set using the characteristics described by Bannister and Hopkins[41]. Additional examples of how features were identified for rendering are shown in S3 Figure.

### Apical end development

**Fig 6:**
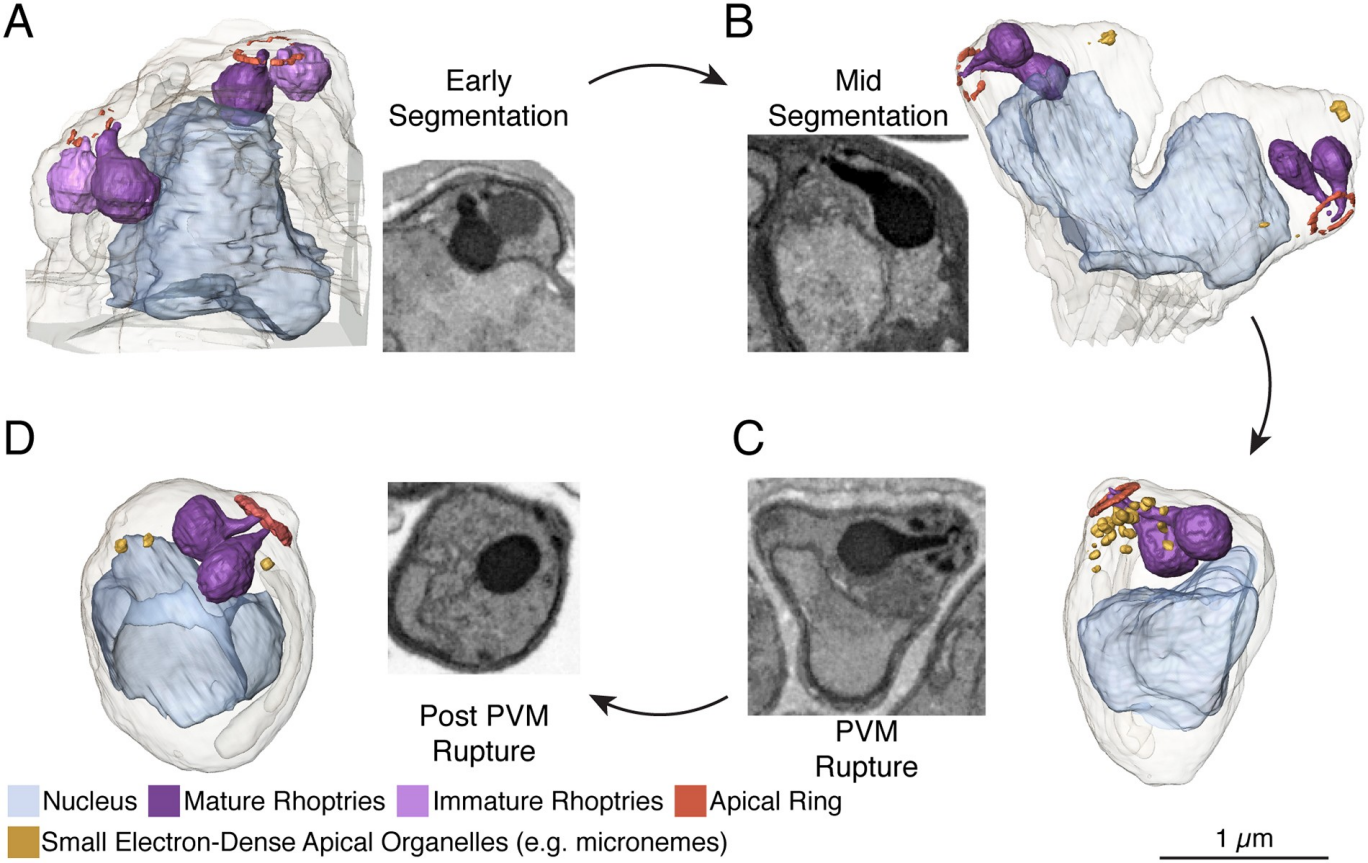
Apical end development throughout schizogony. **A.** Apical end of a 2n nucleus in early segmentation. At this stage, the apical ring is not well-defined, resulting in fragmented rendering. Each pair of rhoptries has a mature, electron-dense bulb, and a less mature, less electron-dense bulb, visible in the electron micrograph inset and rendered in darker and lighter shades of purple, respectively. **B.** At mid-segmentation, the apical ring is more defined and both rhoptries in each set are mature. A few small, electron-dense apical organelles begin to appear. **C.** At PVM rupture, the apical head of the parasite is mature, with a well-defined apical ring, multiple small electron-dense organelles, and mature rhoptries. **D.** Post-PVM rupture, fewer apical organelles remain, presumably due to discharge of a subset of microneme-related organelles, but the defined apical ring and rhoptries are not materially different from the PVM rupture schizont. The three apical polar rings are visible in electron micrographs for each daughter cell, but the first two are too thin to be rendered. Electron micrograph insets for A-C do not correspond to the rendered apical head. Scale bars of renderings interpolated from structure sizes in EM data.

By the beginning of segmentation, rhoptries are visible at each apical bud. At this stage (Fig 6A), one rhoptry is more mature than the other at each bud–judged in EM data by being less electron-dense. Interestingly, we observe that in the rendered parasites, the bulb of the majority of mature rhoptries is in close association with a nearby nucleus (Fig 6A, S1 Table). This suggests a link between rhoptry and apical end formation and the nucleus, as observed in earlier EM studies of rhoptry formation[6], but it is also formally possible that it is an artifact of dehydration and resin embedding for EM preparation. Rhoptry necks reach up into the empty center of the apical polar rings, where three individual apical polar rings are eventually visible for each bud at the end of segmentation. Interestingly, while nearly all apical heads observed contained two rhoptries, each rendered early segmentation schizont contained one to two apical heads with an additional small, electron-dense rhoptry of unclear significance (S1 Table)[6]. At this early stage, no small electron-dense organelles (micronemes, exonemes, dense granules) are visible, concomitant with immunofluorescence studies that show microneme-resident proteins appear only in mature schizonts [42, 43]. By mid-segmentation (Fig 6B), both rhoptries reach their final electron opacity. In some cases the association between the rhoptry and nucleus remains, but for the remaining rhoptry pairs there is no longer a visible nuclear connection (S1 Table). In the mid-segmentation schizonts rendered, one (parasite 2) contained an apical bulb with a third small rhoptry and also contained a pair of miniature rhoptries associated with a nucleus but not an apical bud (S2 Figure). Parasite B had an apical head with a single rhoptry reaching into its apical bud (S1 Table). These results suggest that the vast majority of apical buds form with high fidelity, but that there is a low rate of aberrant bud formation. At mid-segmentation a few small electron-dense organelles are visible, presumably forming micronemes (or exonemes[44], mononemes[45] or other related structures), and dense granules. Finally, at PVM rupture (Fig 6C), the apical end has fully matured, and many small electron-dense organelles fill the space between the rhoptries and the parasite plasma membrane near the apical end of the parasite. Interestingly, in some merozoites, at this stage the apical head of the parasite faces the side, rather than the center top, of the daughter cells. This may be due to physical space constraints within the PVM, as in post-PVM rupture parasites (Fig 6D), the apical organelles reach to the center of the “lemon” (as described by Bannister *et al*.[7]), shaped merozoite. In post-PVM rupture merozoites, fewer small electron-dense organelles are visible, presumably because they discharged while the merozoite was trapped in the RBC.

### The basal end and food vacuole

**Fig 7:**
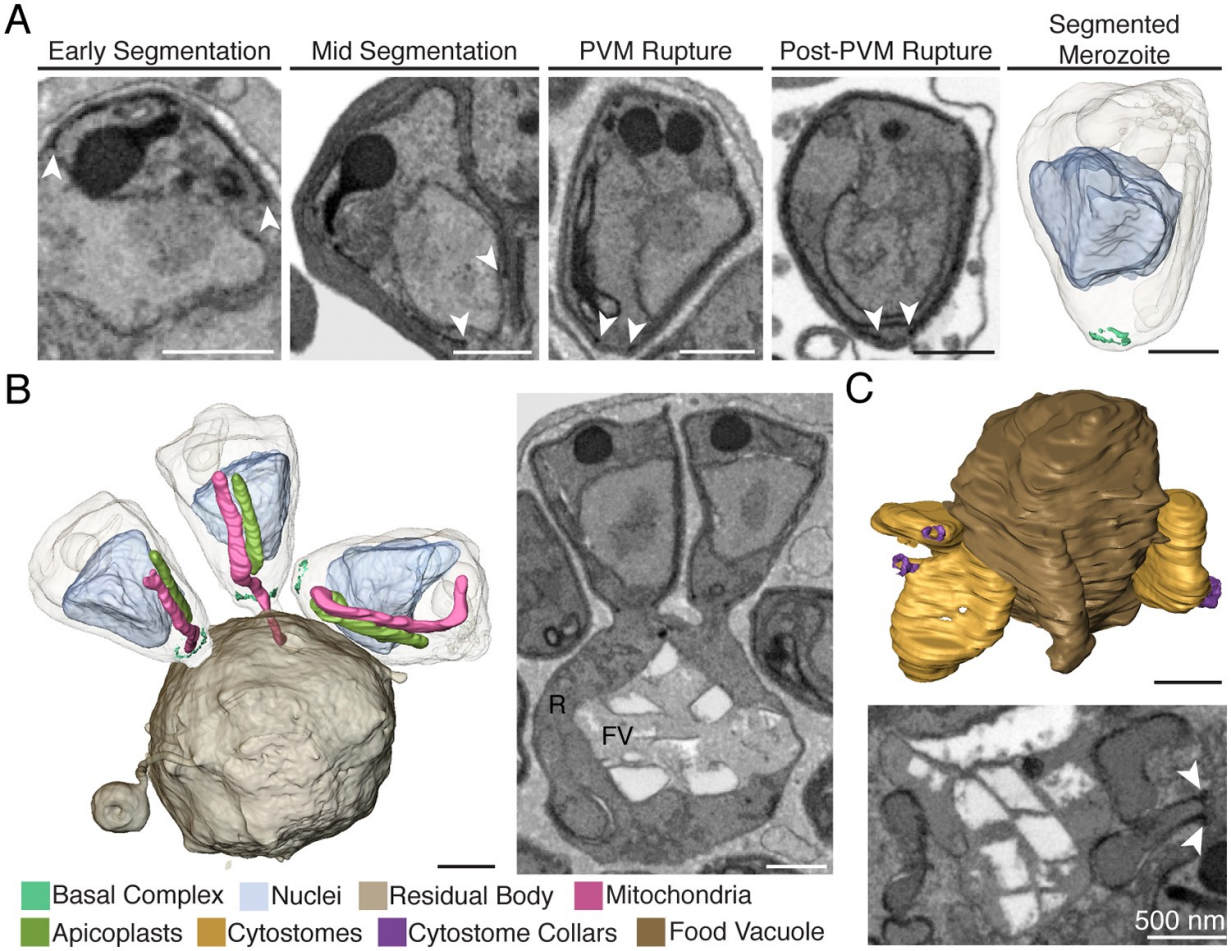
The basal end and food vacuole. **A.** The basal ring resides at the teardrop shaped basal end of the IMC (white arrows). At PVM rupture, this structure is defined enough to render. **B.** At PVM rupture, eight merozoites remained attached to the residual body. Two attached and one unattached merozoites are rendered, including one merozoite with its mitochondrion connected to the residual body. R = residual body, FV = food vacuole. **C.** At mid-segmentation, we visualize the parasite taking up RBC cytosol at cytostomes. These invaginations are located near the food vacuole and their necks are encircled in cytostomal collars, delineated by an electron-dense ring (white arrows). Scale bars of renderings interpolated from structure sizes in EM data.

As membranes push down (or are pulled down) around daughter cell buds to separate individual merozoites, the basal edge of the inner membrane complex is visible as an electron-dense opacity at the posterior end of the IMC (Fig 7A). Here, the basal complex, or contractile ring, is hypothesized to reside, where it likely drives division of daughter cells[38, 46, 47]. This density is visible at all stages but does not have a strong enough signal through our SEM slices to be reliably rendered until late in segmentation (Fig 7A). At this stage the ring sits just inside the bottom of the merozoite’s plasma membrane.

Throughout segmentation, the parasite’s food vacuole resides near the center of the parasite’s mass and is pushed to one edge as if along the backbone of the schizont. In fully segmented schizonts, the food vacuole is in the center of the parasite mass, which we hypothesize is due to membrane tension on the vacuole from the outward protrusion of the surrounding daughter cells. Interestingly, we observe that even at the time of PVM rupture, eight daughter cells remained attached to the residual body (Fig 7B), suggesting that daughter cell fission from the vacuole occurs relatively late in the process of segmentation. Again, it is formally possible, however, that this observed state is an artifact of E64 treatment. We were unable to capture a cell at this stage in the [–]E64 treated sample.

Finally, we observe cytostomes as late as mid-segmentation, with invaginations of red blood cell material residing near the food vacuole (Fig 7C, S1 Table). The cytostomal ring is present around the neck of each invagination as a visible electron-dense intensity. This echoes observations made by Bakar *et al*. in their examination of *P*. *falciparum* morphology by reconstruction of serial electron tomography slices[20].

### Putting the pieces together

**Fig 8:**
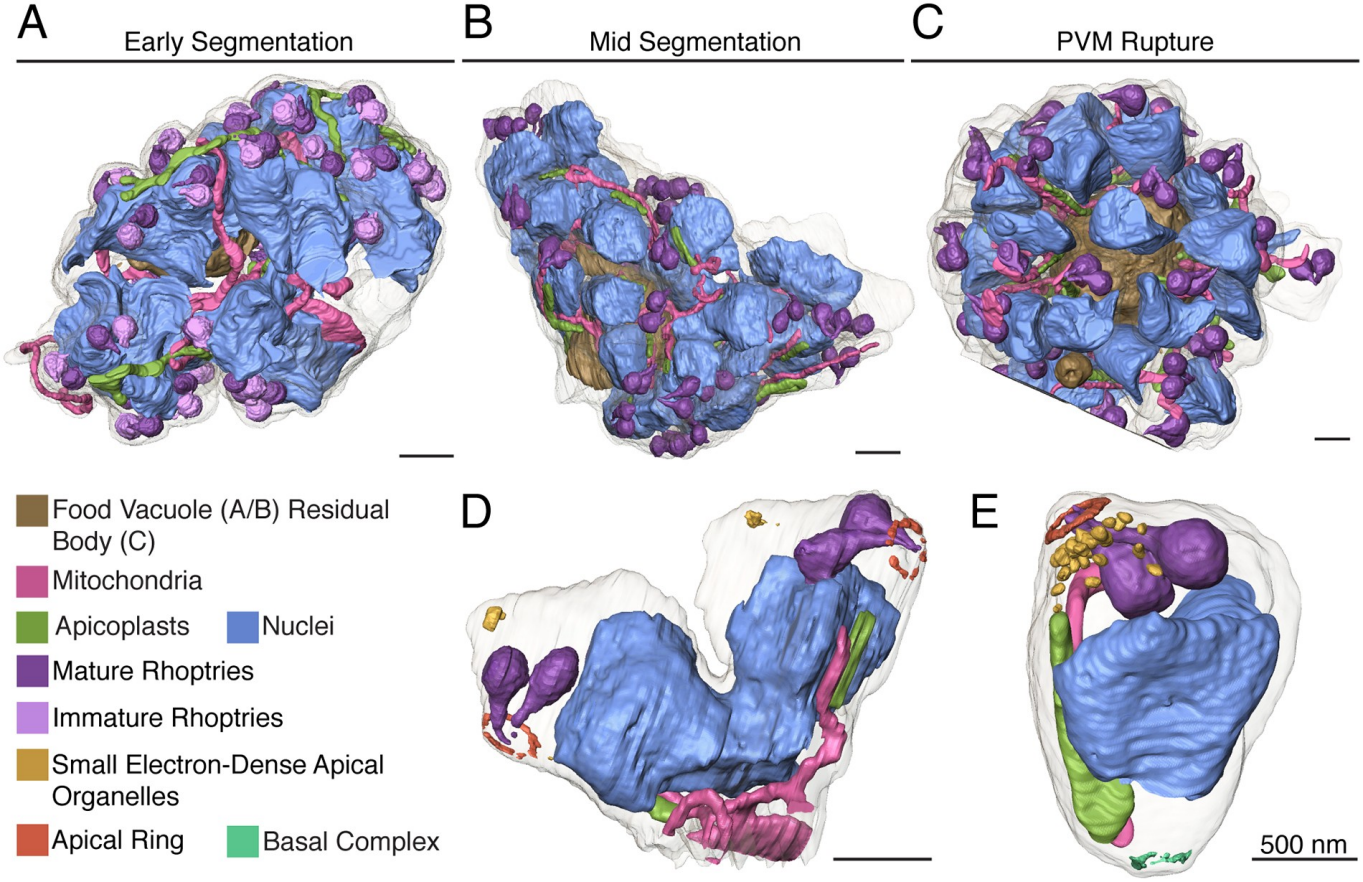
The whole schizont. **A.** In early segmentation, sets of rhoptries composed of one mature and one immature bulb are associated with 4n or 2n nuclei. The apicoplast and mitochondrion twist through the cell, undivided. **B.** At mid-segmentation, pairs of rhoptries are fully mature and nuclei are either 2n or 1n. Apicoplasts are divided and associated with the undivided mitochondrion. **C.** At PVM rupture, individual sets of rhoptries, nuclei, apicoplasts, and mitochondria are separated into their respective merozoite plasma membranes. Most merozoites are fully separated, but eight remain attached to the residual body. **D.** A mid-segmentation bud with material for two daughter cells. The mitochondrion snakes out of the rendered bud to connect with other budding cells. **E.** A fully segmented merozoite with a mature apical head, a segmented nucleus, apicoplast, mitochondrion, and the basal ring. Scale bars of renderings interpolated from structure sizes in EM data.

Over the course of schizogony, the parasite divides its daughter cells by driving membranes around the components for individual merozoites (Fig 8). Although membrane invagination and basal ring formation appears synchronous, the final round(s) of nuclear division appear from our data to be asynchronous. Nuclei divide either from 4n to 2n, then ultimately, to 1n, or from 2n to 1n asynchronously. Apicoplasts, then mitochondria, divide from a serpentine structure to produce one of each organelle per daughter merozoite, rhoptries mature, and small electron-dense apical organelles are formed. The basal end of the parasite closes off around the newly separated organelles, ultimately releasing them from the residual body. The daughter cell is then further freed by egress, where it can invade a new cell to initiate another round of growth and division. The serial sections for each of the rendered schizonts are available as mrc files from the Electron Microscopy Public Image Archive[39] (EMPIAR, Accession EMPIAR-10392, https://www.ebi.ac.uk/pdbe/emdb/empiar/entry/10392/). These files can be viewed with the open source ImageJ software. Movies of the 3D renderings for early segmentation schizont 1, mid segmentation schizont 1, and the PVM rupture schizont are provided as S1–S3 Movies, respectively.

## Discussion

The initial observations of parasite morphology made by parasitologists and electron microscopists over the past 50+ years with both single sections and reconstructed serial sections were strikingly accurate, largely without the use of powerful rendering software. However, FIB-SEM and other volume EM techniques provide three-dimensional cellular context for how organelles and ultrastructural elements relate to one another. In this study, we used FIB-SEM to show the three-dimensional ultrastructure at four stages of *Plasmodium falciparum* segmentation, demonstrating the morphology of the parasite plasma membrane, nuclei, apicoplasts and mitochondria, apical organelles, and basal end at these points in the parasite life cycle. Our data largely complement prior electron and fluorescence microscopy studies that demonstrate the geometry and ultrastructure of the parasite and its organelles and the localization of specific proteins within these organelles, respectively, providing a three-dimensional atlas of the complex process of schizogony. Importantly, using this data we show a novel facet of *Plasmodium* nuclear division. Prior to cytokinesis, the parasite produces components for a variable number, generally 20–36, daughter cells. (In our data, we observe that 3D7 *P*. *falciparum* parasites produce the material for 20 to 36, with a median of 30, daughter cells.) Production of nuclei during this earlier stage has been shown to be asynchronous–instead of dividing from one, to two, to four (and so on), timing of replication initiation varies from nucleus to nucleus, resulting in odd numbers of nuclei throughout early schizogony[4, 28, 48]. Despite this asynchrony through early schizogony, the final round of nuclear division has been hypothesized to be synchronous and simultaneous with cytokinesis[5]. Here, using 3D reconstructions of schizonts from FIB-SEM data, we instead observe asynchronous nuclear division throughout cytokinesis for the first time, suggesting that the final round of nuclear division is also asynchronous. Given that we do observe an even number of nascent merozoites throughout segmentation, we hypothesize that at the initiation of cytokinesis nuclei do undergo one final round of karyokinesis, but this division does not occur synchronously.

As asynchrony of nuclear division through cytokinesis was observed in a handful of dividing parasites, future imaging work should be performed with different conditions (fixation and treatment methods) and modalities (live and fixed fluorescence microscopy) to confirm these results. It is also important to note that here, nuclear division is determined by the status of the nuclear envelope, while fluorescence-based studies of nuclear division rely on DNA intercalating dyes that stain instead for DNA content or antibodies against basal ring proteins[4, 47]. Therefore, to gain a comprehensive view of nuclear division relative to DNA replication, markers for both the nuclear envelope and DNA content will be required.

As for schizogony as a whole, several questions remain about this dynamic process, a few of which are: 1) Are daughter buds pushed outward from the parasite interior, or does the parasite plasma membrane push inward around nascent merozoites? 2) How does the parasite decide when to stop nuclear division and initiate segmentation, and why do we see the nuclear material for up to four parasites dividing when the final number of merozoites is often not a multiple of four? 3) How are organelles directed into individual buds with such high fidelity? (We did not observe any daughters that lacked any of the organelles discussed here.) 4) What cascade results in fission of the merozoites from the food vacuole? 5) For each of these processes, and more not discussed here, what proteins are required for their completion, and, ultimately, what is the mechanistic basis for their function?

Answering these questions will require a comprehensive set of available tools in the parasite, as well as likely yet-to-be developed techniques. Fortunately, we are in a period of burgeoning growth in parasite genetics, cell biology, and the functional evaluation of critical biological processes at a molecular level. The development of tools for both inducible knockdown[49–51] and knockout[52, 53] systems for parasite cell biology and of CRISPR-Cas9 gene editing[54, 55] in the parasite allow us to assess protein localization, function, and essentiality more rapidly than ever before. Building off of these genetic techniques, advanced imaging techniques–ranging from super-resolution microscopy to volume electron microscopy have had and will continue to have an important role in allowing us to visualize the status of both wild-type and transgenic parasites. Resolving the above questions will require both these genetic and imaging techniques. First and foremost, however, key protein players responsible for many of these processes have not yet been identified in *Plasmodium*. Discovering these proteins, and further, characterizing their functions, will be required to ultimately understand the molecular processes driving segmentation.

Given the application of these new tools to parasite cell biology, we are excited to see how our view of the intricate process of schizogony evolves as new data are produced. We hope that the data presented in this study aid the visualization of this complicated process and provide a renewed appreciation for the complex three-dimensional geometry of merozoite formation, thus inspiring future studies of schizogony.

## Materials and methods

### FIB-SEM sample preparation

*P*. *falciparum* schizonts were synchronized two cycles (four days) prior to collection. 75mL of 2% schizonts at 4% hematocrit (HCT) were purified on a 60% percoll gradient. The schizont layer was added to 10mL of media at 4% HCT and allowed to reinvade for two hours, then sorbitol purified to obtain approximately 0.5–1% 0-2h old rings. Parasites were expanded to 20mL at 2% HCT and allowed to egress and reinvade for one cycle. When parasites were 44-46h old (determined by initial Percoll / sorbitol timing), schizonts were magnet purified, eluted, and resuspended in 6mL media. Parasites were aliquoted into 3 wells, two of which were treated with 10μM E64 to prevent egress, and parasites were gassed and put at 37C to mature. Untreated parasites were collected one hour after plating (46-48hpi), and treated parasites were collected three (48-50hpi) and five (50-52hpi) hours post-plating. At the time of collection, parasites were transferred into Eppendorf tubes and pelleted for 5’ at 1850 rpm. The parasite pellet was washed with 1mL incomplete RPMI, resuspended in 50uL incomplete RPMI, and 50uL fixative (2.5% paraformaldehyde, 5% glutaraldehyde, 0.06% picric acid in 0.2M cacodylate buffer) was added to the pellet. Fixed pellets were submitted to the Harvard Medical School Electron Microscopy Core. There, they were washed once in 0.1M cacodylate buffer, twice in water, and then incubated for one hour in 1% osmium tetroxide/ 1.5% potassium ferrocyanide in water. Next, the samples were washed twice in water then once in 50mM maleate buffer pH 5.15 (MB). Samples were incubated for one hour in 1% uranyl acetate in MB, then washed once in MB and twice in water. Pellets were then subjected to dehydration by increasing concentrations of ethanol and were put successively in 50%, 70%, 90%, 100%, 100% ethanol for 10 minutes each. Following dehydration, samples were incubated in propylene oxide for one hour, then overnight with a 1:1 mixture of propylene oxide and TAAB 812 Resin (https://taab.co.uk, #T022). The next day, samples were embedded in TAAB 812 Resin then polymerized at 60C for 48 hours. Between steps, parasite pellets were resuspended in solution then re-pelleted by spinning at 1850rpm for 5 minutes. This staining produced enough contrast to successfully visualize the ultrastructure of sub-cellular features. Thin sections from the 46-48hpi (untreated) pellet and 48-50hpi pellet were stained and visualized on a JEOL 1200EX transmission electron microscope to determine which was best for FIB-SEM analysis. The 50-52hpi pellets consisted primarily of E64 trapped schizonts, which were too fragile to survive sample preparation. Of the two conditions, the 48-50hpi E64-treated pellet and the 46-48hpi untreated pellet contained schizonts of the desired stage range at a density suitable for FIB-SEM and were thus chosen for downstream analysis. To prepare each block for FIB-SEM, the sample region was exposed with a microtome, the epoxy block was mounted onto a standard SEM stub, and the cut surface was rendered conductive via a 5-10-nm-thick sputter-coated Pt/Pd film.

### Data acquisition

Data from the 48-50hpi pellet were collected on a Zeiss Crossbeam 550. The near-surface region was inspected using an external backscatter detector at 20kV and a selected area of 26x26 μm^2^ was covered with a 1–2 μm thick carbon bilayer via ion-beam assisted deposition in the FIB. Sandwiched in between the two layers were fiducial line markings prepared with the ion-beam and filled with Pt. These fiducials were used by the Atlas control software to monitor milling rates and corresponding slice thicknesses during the imaging run. After clearing material in front of the region of interest to a visual depth of 20 μm, data were recorded using the energy-selective back-scatter detector with the electron beam operating at 1.5kV and 2nA and scanning with a pixel dwell time of 8 μs. Milling was performed with the ion beam set to 30kV and a current of 700pA. The raw data set, recorded over a two-day period, contained 1000 images with an approximate pixel resolution of 5nm and a slice thickness of 20nm.

Data from the 48-50hpi pellet of the non-E64 treated parasites were collected on an FEI Helios Nanolab 660. The near-surface region was inspected using an external backscatter detector at 20kV and a selected area of 27x18 μm^2^ was covered with a 1–2 μm thick carbon bilayer via ion-beam assisted deposition in the FIB. As with the Zeiss Crossbeam run, fiducial line markings were prepared with the ion-beam and filled with Pt. After clearing material in front of the region of interest to a visual depth of 20 μm, data were recorded using the in-column back-scatter detector with the electron beam operating at 2kV and 1.6nA and scanning with a pixel dwell time of 4 μs. Milling was performed with the ion beam set to 30kV and a current of 2.5nA. The raw data set, recorded over a two-day period, contained 886 images with an approximate pixel resolution of 5nm and a slice thickness of 20nm. The Pt fiducials were used to carefully align the images during post processing. However, in the Helios run, these fiducials could only be used reliably for the first half of the series (due to limitations of the scan generator). From that point onward, alignment was done by image-to-image cross correlation (correcting short range shifts and shifting the images corresponding to the expected long-range movement).

### Data analysis

Images collected from each run were aligned via cross-correlation, denoised with the non-local means filter, and binned using an in-house Matlab-based software package yielding volumes with a voxel size of 10 x 10 x 20 nm^3^. Analysis scripts available upon request (through corresponding author). In the volumes acquired, we captured approximately 79 partial schizonts (acquisition began mid-cell or ended mid-cell, or the cell was not fully within the boundary of the imaged region; number is approximate because partial cells visualized at the edge of the block could connect in the non-imaged region) and 23 full schizonts in the 48-50hpi block and approximately 75 partial and 15 full schizonts in the 46-48hpi block. Full schizonts at the desired stages were preferentially selected for further analysis, followed by partial schizonts at the desired stage and with the most optimal contrast to aid downstream processing. Early segmentation schizonts were defined as having the beginnings of an apical bud forming around rhoptries and where one rhoptry was more mature (electron dense) than the other. Mid-segmentation schizonts were defined by having membrane invaginations that reached, but did not fully contain, nuclei of the developing daughters and where the width of the open basal end of the invaginating membranes was >500 nm. Fully segmented schizonts were defined as having invaginating membranes that reached 2/3 or more of the nucleus and had an opening at the basal end of <500 nm. The four fully rendered parasites shown in Figs 2, 3 and 5–8 were chosen from the [+]E64 block as follows. The early segmentation schizont was chosen for rendering due to a membrane puncture in sample processing that led to a light space between the PVM and RBC. This increased contrast significantly aided manual segmentation. The mid-segmentation schizont was selected for rendering due to its stage and contrast, although the run ended without capturing the last few sections of the cell. The PVM rupture schizont was the only cell at PVM rupture, but the first few sections of the cell are missing and the left edge of the cell was outside the acquisition boundary. The post-PVM rupture schizont was fully captured and was chosen because merozoite rhoptries had not yet been discharged. The three additional early segmentation and three additional mid-segmentation (one each from the [+]E64 and two each from the [–]E64 block were selected first, because they were fully captured in their respective data sets, and second, for their contrast. The data were then manually segmented and rendered in Avizo 9.2. Nuclei, rhoptries, apicoplasts, mitochondria, small dense apical organelles, cytostomes and cytostome rings, the apical and basal rings, and the food vacuole were identified using previous EM studies as a guide[7]. Nuclear connections for 4n and 2n nuclei were at least 100nm wide through at least 5 Z-sections (100nm deep) to ensure that the observations of connected nuclei were robust. Examples of how organelles were identified are shown in S3 Figure.

## Supporting information

S1 Movie Three-dimensional rendering of a *P*. *falciparum* schizont at early segmentation. Animation of a rendered *P*. *falciparum* schizont at early segmentation. Rendered features (in order of appearance): red blood cell–transparent red, parasite plasma membrane–tan, food vacuole–brown, immature rhoptries–light purple, mature rhoptries–dark purple, apicoplast–green, mitochondrion–pink, and nuclei–blue. At early segmentation, visible buds are present around forming rhoptries. Each rhoptry pair has one more electron dense (mature) and one less electron dense (immature) rhoptry bulb. The apicoplast and mitochondrion are connected, and nuclei are associated with either two apical heads or four apical heads (one representative nucleus is shown for each state at the end).
(M4V)

S2 Movie Three-dimensional rendering of a *P*. *falciparum* schizont at mid segmentation. Animation of a rendered *P*. *falciparum* schizont at mid segmentation. Rendered features (in order of appearance): red blood cell–transparent red, parasite plasma membrane–tan, food vacuole–brown, rhoptries–dark purple, apicoplasts–green, mitochondrion–pink, and nuclei–blue. At mid segmentation, buds are more pronounced. Both rhoptries have reached maximum electron density, apicoplasts are divided, and the mitochondrion is connected. Nuclei are associated with either one or two apical heads (one representative nucleus for each state is shown at the end).
(M4V)

S3 Movie Three-dimensional rendering of a *P*. *falciparum* schizont at PVM rupture. Animation of a rendered *P*. *falciparum* schizont at parasitophorous vacuolar membrane (PVM) rupture Rendered features (in order of appearance): red blood cell–transparent red, parasite plasma membrane–tan, residual body–brown, immature rhoptries–light purple, mature rhoptries–dark purple, apicoplasts–green, mitochondria–pink, and nuclei–blue. At PVM rupture, daughter cells are mostly individualized and contain a pair of rhoptries and a single mitochondrion, single apicoplast, and single nucleus. Some daughter cells (two shown) remain attached to the residual body, while others (one shown) are fully detached.
(M4V)

S1 Table Features of all rendered schizonts at early and mid-segmentation. In this table the features of four rendered early segmentation and four rendered mid segmentation parasites are recorded. Nuclear status was determined by the number of apical buds each nucleus was associated with. Rhoptry pairs were determined to be associated with the nucleus if one of the bulbs made contact with its associated nucleus. Cytostomes were determined to be present if the cytostome, including cytostome ring, was observed within the parasite. Apicoplasts and mitochondria were determined to be divided if the majority of daughter cells contained their own individual respective organelle.
(PDF)

S2 Table Nuclear counts for all non-rendered schizonts at early, mid, and late segmentation. (PDF)

S1 Figure Rendered nuclei and rhoptries for three early segmentation schizonts. Rendered nuclei and associated rhoptries shown for early segmentation schizont 2 (from [+]E64 sample) and early segmentation schizonts A and B (from [–]E64 sample). A small connection is visible between two of the 1n nuclei in schizont 2 –this did not meet our parameters of at least 100nm wide and 100nm deep to be counted as a connection, therefore each bulb was counted as a 1n nucleus. Interpolation of scale bars not performed for these renderings.
(TIF)

S2 Figure Rendered nuclei and rhoptries for three mid-segmentation schizonts. Rendered nuclei and associated rhoptries shown for mid segmentation schizont 2 (from [+]E64 sample) and mid segmentation schizonts A and B (from [–]E64 sample). For mid-segmentation schizont 2, the rhoptry set with a third small bulb can be seen in the center of the 1n image. Additionally, the pair of miniature rhoptries not associated with an apical bud can be observed on the right-most nucleus of the same image. Interpolation of scale bars not performed for these renderings.
(TIF)

S3 Figure *P*. *falciparum* ultrastructural features. Identification of several of the *P*. *falciparum* organelles rendered in this study. **A.** Selected region of the PVM rupture parasite. **B.** Selected region of the mid-segmentation schizont. **C.** Selected region of the early segmentation schizont. **D.** Selected merozoites from the post-PVM rupture schizont. For all images rhoptries (Rhop), mitochondria (Mito), apicoplasts (Ap), and nuclei (N) identified.
(TIF)
