# Supplementary material for: Three-dimensional ultrastructure of Plasmodium falciparum throughout cytokinesis
Source: PLoS Pathog. 2020 Jun 8;16(6):e1008587. doi: 10.1371/journal.ppat.1008587 (PMC7302870; doi:10.1371/journal.ppat.1008587)
Supplement: S1 Table — In this table the features of four rendered early segmentation and four rendered mid segmentation parasites are recorded. Nuclear status was determined by the number of apical buds each nucleus was associated with. Rhoptry pairs were determined to be associated with the nucleus if one of the bulbs made contact with its associated nucleus. Cytostomes were determined to be present if the cytostome, including cytostome ring, was observed within the parasite. Apicoplasts and mitochondria were determined to be divided if the majority of daughter cells contained their own individual respective organelle. (PDF) [file ppat.1008587.s004.pdf]

	Rendered Parasite	Nuclei				Total Mero	Rhop assoc/ nonassoc	Cyto-stomes Present?	Apicoplast Divided?	Mito Divided?
		4n	2n	1n	Partial					
**Early Segmentation**	Schizont 1 [+] E64	2	11	0	0	30	30 / 0	No	No	No
	Schizont 2 [+] E64	0	10	4	0	24	23 / 1	Yes	No	No
	Schizont A [-] E64	0	12	0	0	24	24 / 0	Yes	No	No
	Schizont B [-] E64	0	15	0	0	30	25 / 5	Yes	No	No
**Mid Segmentation**	Schizont 1 [+] E64	0	5	13	5	?	14 / 14	Yes	Yes	No
	Schizont 2 [+] E64	0	7	18	0	32	26 / 6	Yes	Yes*	No
	Schizont A [-] E64	0	6	22	0	34	25 / 9	Yes	Yes	No
	Schizont B [-] E64	0	10	12	0	32	24 / 8	Yes	Yes	No

	Rendered Parasite	Notes
**Early Segmentation**	Schizont 1 [+] E64	Two apical heads with three rhoptries. 4n nuclei connected by branches of at least 100nm in XY over at least 5 Z-slices (100nm)
	Schizont 2 [+] E64	One apical head contains a third small rhoptry. There is a small lobe of nuclear material (~600nm in diameter) not associated with any apical heads.
	Schizont A [-] E64	One apical head contains a third small rhoptry.
	Schizont B [-] E64	One apical head contains a third small rhoptry.
**Mid Segmentation**	Schizont 1 [+] E64	Partially captured parasite (run ended before end of cell was captured – can't predict total number of merozoites)
	Schizont 2 [+] E64	One nucleus is associated with an additional miniature pair of rhoptries that are not associated with an apical bud. One apical head contains a third small rhoptry. *One apicoplast is shared between two buds.
	Schizont A [-] E64	None
	Schizont B [-] E64	One apical head with a single rhoptry present
