# Supplementary material for: Three-dimensional ultrastructure of Plasmodium falciparum throughout cytokinesis
Source: PLoS Pathog. 2020 Jun 8;16(6):e1008587. doi: 10.1371/journal.ppat.1008587 (PMC7302870; doi:10.1371/journal.ppat.1008587)
Supplement: S2 Table — (PDF) [file ppat.1008587.s005.pdf]

	**Analyzed Parasite**	Nuclei			Total Mero
		4n	2n	1n	
**Early Segmentation**	Schizont 3 [+] E64	0	13	0	26
	Schizont 4 [+] E64	0	10	0	20
	Schizont 5 [+] E64	0	11	0	22
	Schizont 6 [+] E64	0	15	0	30
	Schizont C [-] E64	1	14	2	34
**Mid Segmentation**	Schizont 3 [+] E64	0	3	22	28
	Schizont 4 [+] E64	0	0	24	24
	Schizont C [-] E64	0	9	12	30
	Schizont D [-] E64	0	6	18	30
**Late Segmentation**	Schizont 1 [+] E64	0	0	36	36
	Schizont 2 [+] E64	0	0	30	30
	Schizont 3 [+] E64	0	0	22	22
	Schizont A [-] E64	0	0	32	32
