# Supplementary figures and images for: Three-dimensional ultrastructure of Plasmodium falciparum throughout cytokinesis
Source: PLoS Pathog. 2020 Jun 8;16(6):e1008587. doi: 10.1371/journal.ppat.1008587 (PMC7302870; doi:10.1371/journal.ppat.1008587)

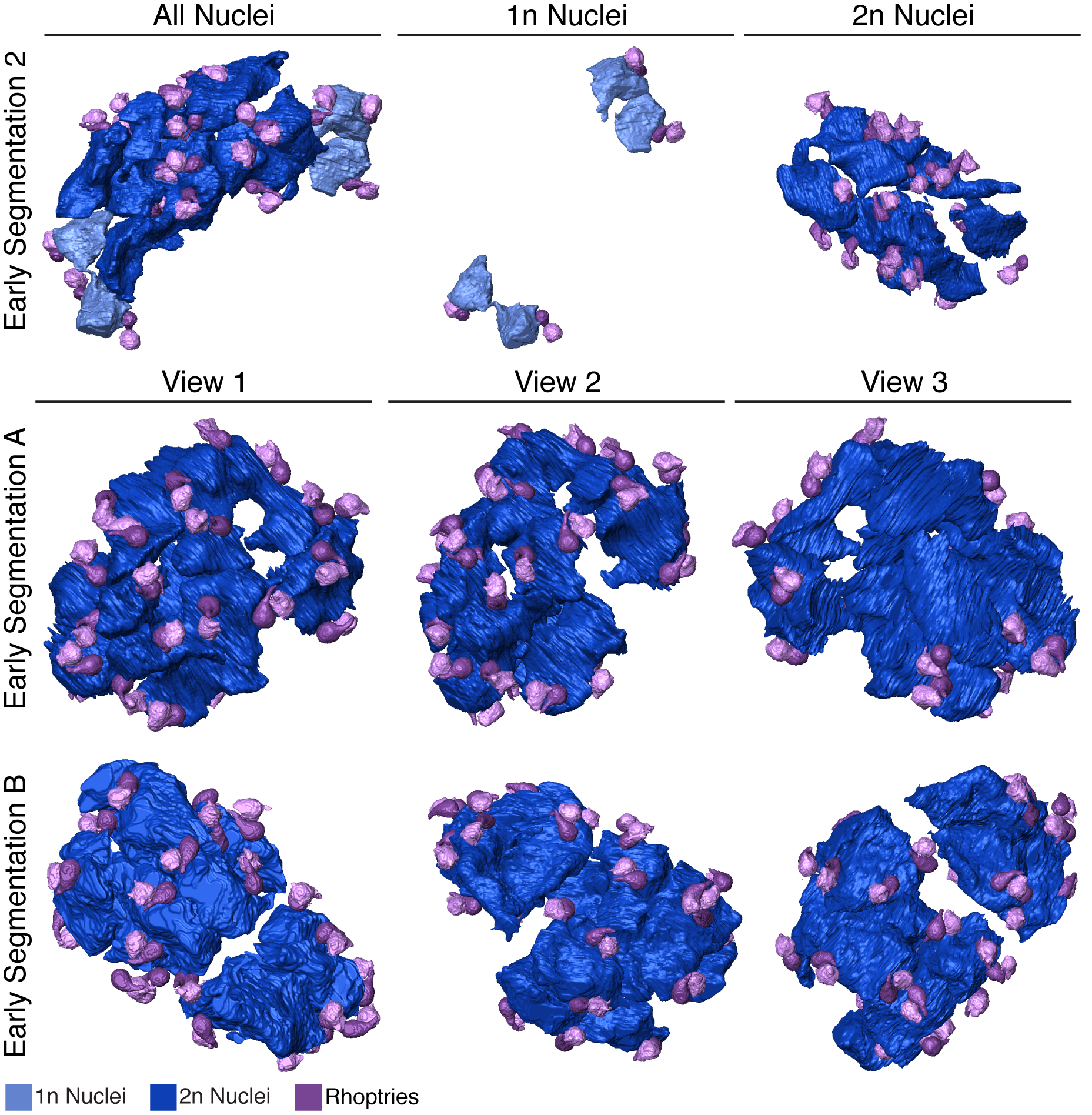

Supplement: S1 Figure — Rendered nuclei and associated rhoptries shown for early segmentation schizont 2 (from [+]E64 sample) and early segmentation schizonts A and B (from [–]E64 sample). A small connection is visible between two of the 1n nuclei in schizont 2 –this did not meet our parameters of at least 100nm wide and 100nm deep to be counted as a connection, therefore each bulb was counted as a 1n nucleus. Interpolation of scale bars not performed for these renderings. (TIF) [file ppat.1008587.s006.tif]

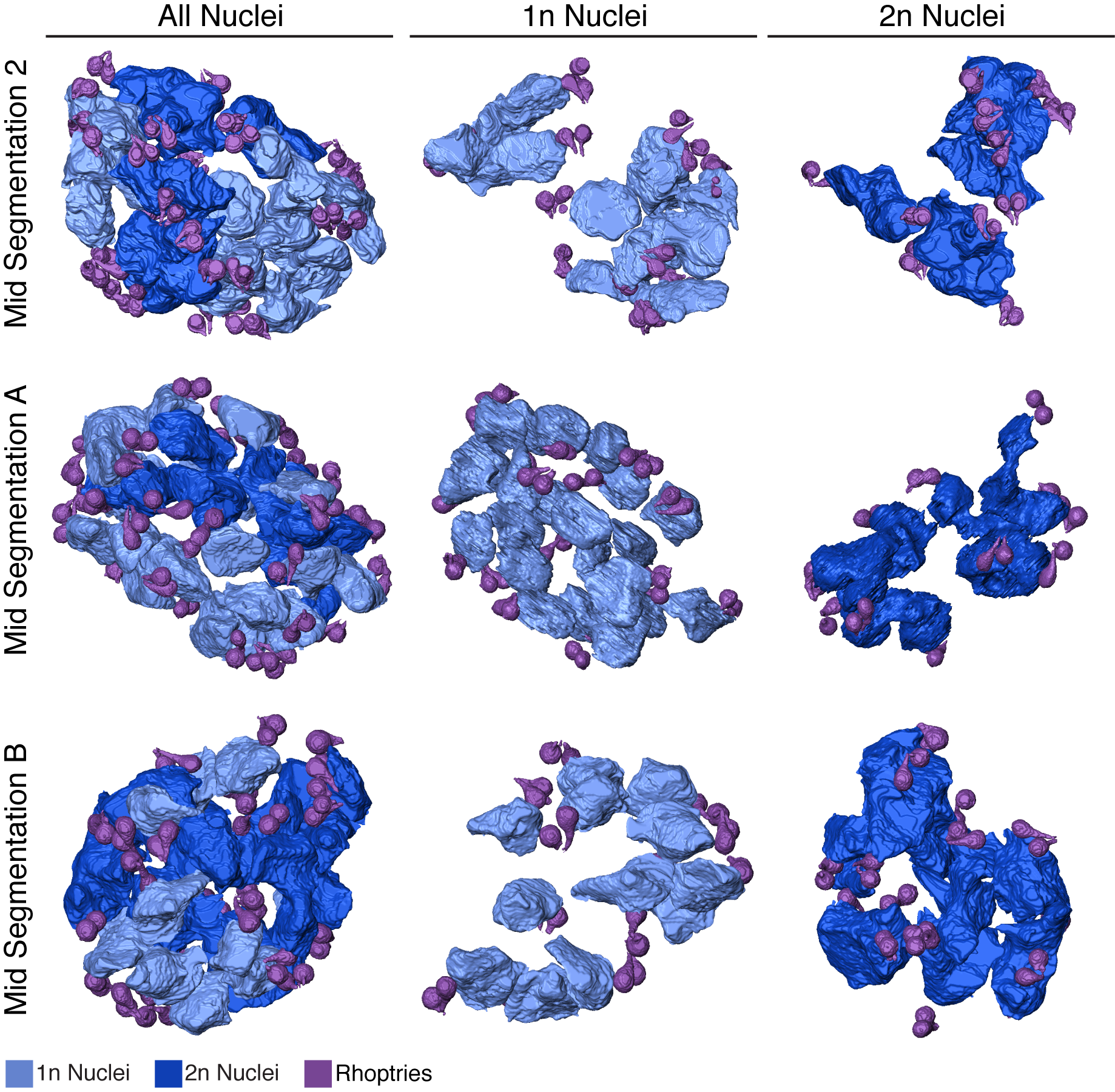

Supplement: S2 Figure — Rendered nuclei and associated rhoptries shown for mid segmentation schizont 2 (from [+]E64 sample) and mid segmentation schizonts A and B (from [–]E64 sample). For mid-segmentation schizont 2, the rhoptry set with a third small bulb can be seen in the center of the 1n image. Additionally, the pair of miniature rhoptries not associated with an apical bud can be observed on the right-most nucleus of the same image. Interpolation of scale bars not performed for these renderings. (TIF) [file ppat.1008587.s007.tif]

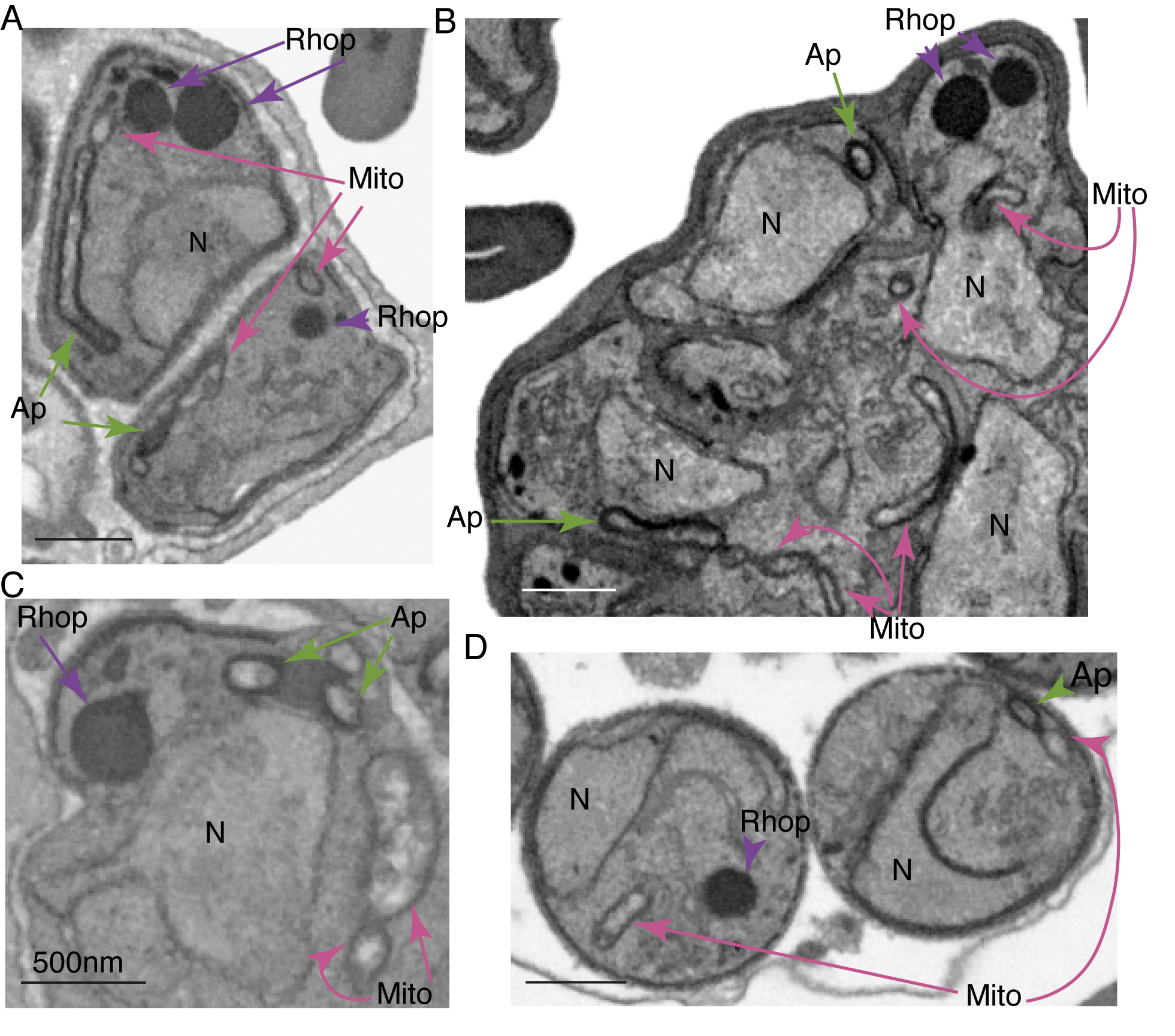

Supplement: S3 Figure — Identification of several of the P. falciparum organelles rendered in this study. A. Selected region of the PVM rupture parasite. B. Selected region of the mid-segmentation schizont. C. Selected region of the early segmentation schizont. D. Selected merozoites from the post-PVM rupture schizont. For all images rhoptries (Rhop), mitochondria (Mito), apicoplasts (Ap), and nuclei (N) identified. (TIF) [file ppat.1008587.s008.tif]
